# The Kata-Thermometer

**Published:** 1919-06-14

**Authors:** 


### THE KATA-THERMOMETER.

METEOROLOGICAL records, as hitherto taken, have had much more significance for the physicist than for the physiologist, and for the mariner than for the medical man. Tables of maximum and minimum temperatures, of the duration of sunlight, of absolute and relative humidity, such as are published by meteorological bureaus, and advertised by rival health resorts, do not throw much light on weather and climate in their bearing on health and physiological functions; for the body is not exposed to these separately, nor are its reactions to varying meteorological environment purely passive. For a good many years indeed, physiologists and climatologists have begun to realise that the most important health factors of climate are not the temperature or humidity of the air, but its dual capacity for cooling the body and for promoting evaporation from it, a capacity depending on the interactions of the temperature, humidity and movement of the air, with the ordinary physiological functions of thermogenesis, glandular secretion, etc.

Energy, whether muscular or chemical, produces heat, and if the body be not efficiently cooled by the air it will curb its energy in order to reduce its heat production, and avoid pyrexia. If the air be insufficiently cooling, energy will flag. Again, if the air be insufficiently drying, health, even apart from the cooling effect of evaporation, will suffer; for a rapid passage of fluid from the skin and mucous membranes is conducive to health in various ways, and any retardation of its passage correspondingly pernicious. Ordinary thermometers and meteorological instruments do not record the cooling and drying value of the atmosphere, and take no account of the movements of the air in promoting both cooling and drying, and it has been necessary to find a suitable instrument to measure these important hitherto unrecorded factors.

Recently, Dr. Leonard Hill has been using for this purpose a thermometer named a kata-thermometer, and at a lecture delivered before the Royal Meteorological Society on March 19, he described the advantages of this instrument. By its means we get exactly what we require—a record of the cooling and evaporative capacity of the air whether still or in movement. The interest and value of such records cannot be over-estimated: they will put climatology on a sound scientific basis, and there can be little doubt that in the not distant future their readings will be the main criteria of climate.

Measured by the kata-thermometer, Aberdeen is found to have a more bracing climate than Kew, and England a more bracing climate than America, and as observations accumulate, it will grow possible to assort climates with a scientific precision never before attained. But, more than this, the records of the kata-themometer will teach the public the hygienic value of breezes and moving air, and perhaps even bring about a much needed reform in domestic ventilation. Experiments made by Dr. Leonard Hill showed that it was quite common for people in England to live in rooms with an atmosphere not unlike the atmosphere of Ceylon, and under such atmospheric conditions, health and energy cannot possibly be fully maintained. The kata-thermometer, therefore, is likely to carry admonition and warning and to be a health measure in a double sense of the term.

